# Team science competencies across the career life course for translational science teams

**DOI:** 10.1017/cts.2024.494

**Published:** 2024-03-08

**Authors:** Angela M. Mendell, Verena Knerich, Damayanthi Ranwala, Carolynn T. Jones, Patricia Piechowski, Catherine W. Striley, Wayne T. McCormack, Jennifer E. Cross

**Affiliations:** 1 Center for Clinical & Translational Science & Training, University of Cincinnati, Cincinnati, OH, USA; 2 Department of Sociology, Colorado Clinical and Translational Sciences Institute, Institute for Research in the Social Sciences, Colorado State University, Fort Collins, CO, USA; 3 Institute of Intercultural Communication, Ludwig Maximilian University, Munich, Germany; 4 Department of Bioengineering, College of Engineering, Computing and Applied Sciences, Clemson University, Clemson, SC, USA; 5 Center for Clinical Translational Science and College of Nursing, The Ohio State University, Columbus, OH, USA; 6 Michigan Institute for Clinical & Health Research, University of Michigan, Ann Arbor, MI, USA; 7 Department of Epidemiology, Colleges of Medicine & Public Health and Health Professions, University of Florida/Florida State University Clinical & Translational Science Institute, University of Florida, Gainesville, FL, USA; 8 Department of Pathology, Immunology and Laboratory Medicine, College of Medicine, UF/FSU Clinical & Translational Science Institute, University of Florida, Gainesville, FL, USA

**Keywords:** Competencies, collaborative research, community partners, life course, professional development, role progression, team science

## Abstract

**Introduction:**

Translational science (TS) teams develop and conduct translational research. Academic TS teams can be categorized under three constituency groups: trainees and faculty, clinical research professionals (CRP), and community partners. Our study objectives were to define individual and team competencies of these three constituency groups during their career life course and determine relative importance and the level of mastery of each of the competencies needed at different stages of their life course.

**Methods:**

Each group was composed of experts for their constituency group. We applied individual and team competencies in TS teams by Lotrecchiano *et al*. (2020) as a starting point for structured expert discussions following a modified Delphi approach that we adapted based on the emergent needs and insights per constituency group.

**Results:**

The degree of relevance and level of mastery for individual and team competencies varies for trainees and faculty members across the career life course based on opportunities provided and relative importance at that career stage. However, CRPs enter TS teams at various career stages with fundamental, skilled, or advanced levels of smart skills that may or may not be contextual to their role. Community partners equally possess and develop competencies in a non-linear and contextual fashion that are required to facilitate constructive, bi-directional collaboration with other members of TS teams.

**Conclusions:**

Team science competencies across the career life course do not develop linearly among different constituency groups and require an adaptive framework to enhance TS team effectiveness.

## Introduction

Team science is an overarching approach to collaborative research that draws from empirical evidence to create the conditions for success on diverse, cross-disciplinary teams by developing, applying, and evaluating strategies, tools, and programs. One manifestation of team science is translational research, where the goal is to move observations from the lab to the clinic and then to the community for the improvement of health in populations [1]. TS includes not only translational research but a deeper understanding of the science and operational principles that improves efficiency, effectiveness, quality, ethics, and application of the research [2–4]. TS teams in academic settings commonly involve three constituency groups, firstly faculty scientists (clinician scientists and PhD scientists) and/or trainees (undergraduate and graduate students, postdoctoral and clinical fellows) who conduct their research in basic, clinical, social science, and community settings. A vast array of clinical research professionals (CRPs) form the second constituency group and are integral to operationalizing various aspects of translational research, including but not exclusively project management, recruitment, informed consent, regulatory affairs, data management, clinical care, pharmacy, community relationships, and grants administration. Thirdly, community partners may be involved, ensuring that project design and a sustainable translation of findings can be accomplished, not for, but with the community concerned [5]. Community partners may therefore come from the setting to which the research is being translated.

Whether TS is performed in a community setting or in a traditional academic or clinical trial setting, all team members need a variety of skills to conduct collaborative research. Researchers and funders alike recognize a growing need for targeted skill acquisition by developing team science training programs. For instance, training the faculty and trainees in team science has been the focus of funded initiatives through the National Center for the Advancement of Translational Science (NCATS) [6–8]. Likewise, multiple researchers have proposed schema that describes the team science approach to conducting pre-clinical, clinical, and community research [5–9]. A recent article on TS described seven fundamental characteristics or skills for translational scientists: boundary crosser, team player, process innovator, domain expert, rigorous researcher, skilled communicator, and systems thinker [10]. Lotrecchiano *et al.* [11] conducted a Delphi study to further define what competencies drive success on TS teams not only on the individual but also on the team level. They defined team science competencies across five domains (Table 1) and identified five specific competencies for individuals and eight competencies for teams. The intersections among individual and team competencies across the domains illustrate the overlapping nature of teaming [11].

**Table 1.**
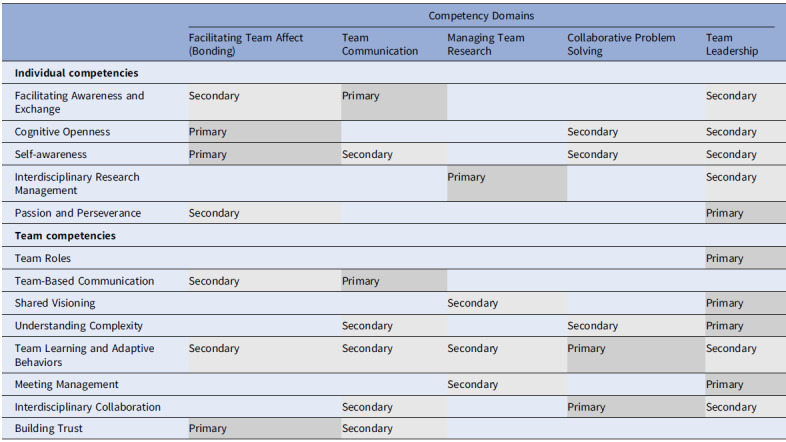
Individual and team competencies, adapted from Lotrecchiano *et al*. [11]

This study builds on the team science competencies developed by Lotrecchiano *et al*. [11] and refines them across the life course for the three distinct constituencies in academic TS teams mentioned previously. Defining different teaming competencies across the life course can help inform new curriculum development and assessment criteria for targeted training and career progression. By comparing different constituency groups, their specific needs and contributions can be addressed through tailored assessment and training. A better understanding of competencies across the life course can therefore enhance performance at Clinical and Translational Science Award (CTSA) hub institutions and similar research centers to enhance innovation, discovery, and the quality of the research [12].

The authors of this study are part of a working group that emerged from the Team Science Education & Training Special Interest Group (SIG) within the International Network for the Science of Team Science (INSciTS). The SIG is a collaborative group that meets via discussion, document, and toolkit sharing through the INSciTS listserv. The working group split into three subgroups that were charged with describing each constituency’s team roles, life course, and outputs developed, in terms of individual and team science skill acquisition. Here we first describe the composition of the subgroups exploring team science competencies for the three constituency groups (trainees and faculty, CRPs, and community partners). We then share details about our methods and present results per subgroup. Our discussion compares findings across all three subgroups to synthesize our insights.

## Materials and methods

### Trainees and faculty constituency group

Members of this group, led by two co-chairs (DR and WTM), were subject matter experts based on their CTSA leadership roles in team science education and training, and TS workforce development. TS teams may include trainees and faculty at all levels and other stakeholders. For this work, we considered trainees and faculty at five stages of career development: undergraduate student (trainee), graduate student (trainee), postdoctoral fellow (trainee), junior faculty (early-stage investigator), and middle-senior faculty (associate professor and professor). Whereas we understand that all the competencies defined by Lotrecchiano, *et al*. [11] play critical roles during the lifespan of trainees and faculty, we wanted to determine the degree of relevance of the competencies for each career stage, when individuals should be learning the competencies, and what level of mastery is needed for them to be an effective team member. We mapped individual competencies and team competencies for individuals at each stage. After initial constituency group meeting discussions, each member independently ranked each of the competencies (Table 2) on a three-point scale from lowest to highest (+ = lowest; +++ = highest) relevance and the level of mastery at each career stage. Rankings were pooled and discussed to reach consensus for a final ranking.

**Table 2.**
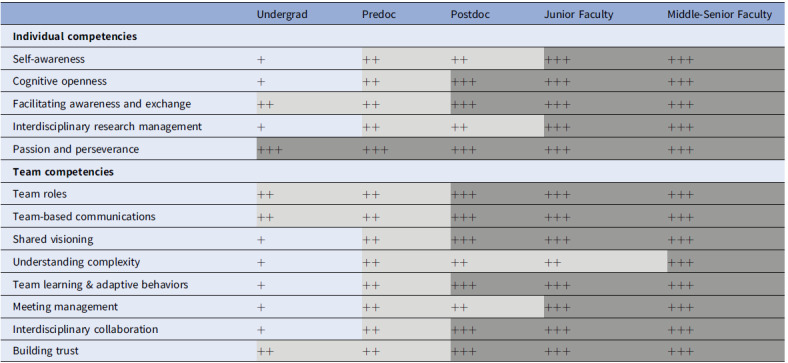
Relevance and level of mastery ranking for individual and team competencies for trainees and faculty

^+^ least level of relevance and mastery.

^++^ moderate level of relevance and mastery.

^+++^ highest level of relevance and mastery.

### CRP constituency group

The CRP constituency group was led by two co-chairs (AM, CJ), who recruited CRPs working at multiple CTSA hub institutions and from a variety of CRP roles to participate, including those working in the team science space. After establishing a project charter and working group norms, we performed an initial literature search on team science for CRPs. The Joint Task Force (JTF) Clinical Trial Competencies version 3.1 lists two domains that overlap with team science: Domain 7: Leadership and Professionalism (4 competencies) and Domain 8: Communications and Teamwork (4 domains) [13,14], however; we realized there was a paucity of published literature on the topic of team science for CRPs at the onset of this endeavor. The lack of literature on the topic of CRPs gave merit to our aims, especially in the current CRP workforce crisis [15].

We adopted and outlined a multi-step Delphi process to define team science competencies for CRPs [16]. Our CRP constituency working group defined CRPs as staff members (non-faculty) whose role is to support the operation and management of translational research activities (including clinical research coordinators, clinical research nurses, quality assurance managers, regulatory affairs managers, administrators, data managers, research pharmacists, lab personnel, among other job titles). We then defined the CRP professional life course. Because there was similarity in progression across roles, the group agreed to use the three stages of professional development informed by Benner (novice to expert) and adopted by the JTF and Duke University as the life course for the CRP profession (e.g., fundamental, skilled and advanced) [14,17]. In the third stage, the 17 CRP collaborators were divided into four groups, with a chair leading each group in a Delphi process to apply individual and team competencies [11] by defining smart skills, which were then ranked as fundamental, skilled, and advanced. As groups rotated through each of the 13 competencies in defined stages, collaborative edits were made and discussed to achieve an agreed complement of leveled CRP team science smart skills.

### Community partners constituency group

This group began with three co-chairs (JC, PP, and CS), who met to establish ground rules and expectations for the team. To discuss the role of community partners in team science, this team reviewed definitions of “community” and “partner” in the field of TS as defined by the CDC Principles of Community Engagement [18]. For our definition of community, we considered both a grassroots and inclusive approach including members of and leaders of grassroot organizations, specific disease advocacy organizations, and nonprofit organizations; community health workers (who often are employed by such organizations); public health professionals; patients and their families; politicians and governmental agency employees; staff, students, and teachers from the educational sector; industry partners; health care professionals of all types; and social workers, neighborhood associations and living communities, and any other interested party. While this definition is not exhaustive, it includes a wide spectrum of community partners engaged in team science that we have seen and imagined at the table.

Next, we expanded the team by inviting new members who represent different types of community partners, a long-time citizen scientist, grassroots community partner, community organizer, deputy director of a university community engagement office, and additional researchers. The team met monthly to read and review the Lotrecchiano *et al*. [11] article and to discuss how the team science competencies overlap with or diverge from the competencies identified for community-engaged research (CEnR) [19–21]. We used an online Miro board to compare across competency frameworks and generate competencies that are missing from existing frameworks for researchers and community partners. From this, we generated new competencies that were added to the existing team science competencies [11] and used them to create specific milestones. Throughout the process, community partners emphasized the mutual learning and personal development needed by community partners and researchers in community-partnered team science and challenged the group to suspend our initial assumptions and expectations.

A core assumption of this process had been that a developmental, linear model for building team science competencies could be applied to the diverse community partners. During this process, our unconscious biases as researchers were challenged to recognize the wide variation in roles of community partners, their preexisting competencies and knowledge, individual motivations for teaming, and their self-defined need for acquiring additional teaming skills. Our own learning was towards a more inclusive practice when working with community partners, in that our paradigm shifted from thinking as educators to thinking as collaborators, appreciating the diversity in life experience and skills of each team member, whether researcher, CRP, or community partner.

## Results

### Trainee and faculty constituency group

Table 2 shows the degree of relevance and level of mastery of each individual competency by career stage. Among the individual competencies, Passion and Perseverance were ranked as highly relevant at all stages of the trainee and faculty career lifespan. At the undergraduate student stage, three of the other four individual competencies were ranked as least relevant (Self-awareness, Cognitive Openness, and Interdisciplinary Research Management) and Facilitating Awareness and Exchange was ranked as moderately relevant, based on their sometimes-limited roles as team members, typically under close supervision and sometimes with little autonomy. For predoctoral trainees, the relative importance was ranked as moderate for all four other individual competencies, given their roles as team members with some supervision, but developing some independence in designing dissertation research projects. For postdoctoral trainees, two competencies (Self-awareness and Interdisciplinary Team Management) were ranked as moderately relevant, and two additional competencies were ranked as highly relevant (Cognitive Openness and Facilitating Awareness & Exchange), given the increased independence that postdoctoral trainees have in their research. All individual competencies were ranked as highly relevant for the junior faculty and middle-senior faculty, given their leadership roles in directing research laboratories and the expectation that they serve as role models and mentors.

All team competencies were ranked as least relevant or moderately relevant for undergraduate students, again based on their usually limited roles as research team members. It was noted that to be effective team members, the competencies of Team Roles, Team-Based Communication, and Building Trust were most relevant, and should be developed early. All team competencies were ranked as moderately relevant for predoctoral trainees, given their central role in many academic research team members, their developing independence, and often their roles in supervising undergraduate students. Most team competencies were ranked as highly relevant for postdoctoral trainees, given their increased independence, roles in supervising and near-peer mentoring of undergraduate and graduate students, and preparation for the next step in their research career. All but one of the competencies were ranked as highly relevant for junior faculty members. The exception was Understanding Complexity, due to the “on the job” training that occurs with the transition to a faculty position. All team competencies were ranked as highly relevant for middle- to senior faculty members, again given their leadership roles in directing research groups, establishing research collaborations with colleagues at their own institution and/or at other institutions, and the expectation that they serve as role models and mentors for trainees at all levels.

### CRP constituency group

The CRP constituency working group ultimately developed 59 smart skills (22 individual and 37 team competency skills) across the five individual and eight team competencies. Each of these was articulated into three levels, fundamental, skilled, and advanced. Table 3 illustrates a sample of three leveled smart skills developed for two individual competencies Facilitating Awareness and Exchange and Interdisciplinary Research Management. By illustrating each through ranked action terms, this work can be applied as a basis for team science awareness, and team science training for CRPs. Moreover, these skills can be applied to ranked job descriptions and be used in performance evaluations to aid in professional development and progression. The full set of CRP Team Science Competencies developed by this group is further described in another article and supplemental materials in that issue [22].

**Table 3.** Illustration of Bloom’s taxonomy applied to clinical research professional (CRP) team science smart skills and leveled competencies

Facilitating awareness and exchange *(Individual Competency)*			
***Defined as:*** *Sharing information and perspectives, active listening, and probing, reframing skills* [11].			
***Smart skills:***	**Fundamental**	**Skilled**	**Advanced**
**Relational openness**	***Recognize*** *the importance of relational openness as a team member*	***Exhibit*** *relational openness by welcoming and introducing team members*	***Create*** *a welcoming, inclusive, and positive environment*
**Awareness of individuals’ points of view**	***Express*** *understanding of other people’s point of view*	***Demonstrate*** *open, flexible perspectives, honoring different points of view*	***Support*** *differences in points of view*
**Interdisciplinary research management** (individual competency)			
***Defined as*** *: Ability to manage diverse and multi-team systems. Develop team skills to strengthen team structures and dynamics* [11].			
**Smart skills:**	***Fundamental***	***Skilled***	***Advanced***
***Respect***	***Acknowledge*** *the importance of respecting your team members*	***Exhibit*** *respect for team members and colleagues via active listening, rapid follow-up, and sensitivity to both verbal and nonverbal communication.*	***Integrate*** *appropriate training to build and promote respectful workplace habits.*

### Community partners constituency group

**Figure 1.**
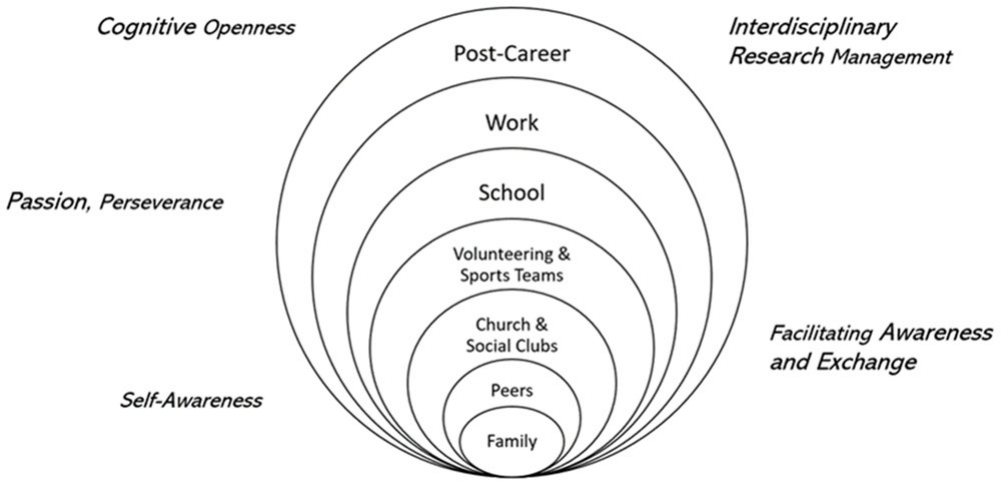
Teaming competencies develop non-linearly across lived experiences.

*Individual competencies develop in a non-linear fashion and across environments.* As this constituency group discussed how team science competencies [11] apply to community-engaged teams, we identified a few ways that these teams are different. First, we realized that the questions that motivated this working group “How do team science competencies develop and change over the life course of a scientist?” and “How should we sequence team science training to consider the developmental trajectory of competencies?” were not well aligned for thinking about community partners. These questions were centered on the perspective of scientists as educators, not as research partners. The diversity of community partners means that on each engaged team, these partners enter teams with varying lived experiences, personal experiences, professional experiences, and diverse experiences in teaming constituting valuable competencies that should be recognized. Rather than the stages that students may move through in developing teaming skills within educational and research environments, community partners are developing these same individual competencies in a variety of environments, and typically not in the contemplated linear fashion (see Fig. 1).

*The identification of opportunities for training of community partners needs to take preexisting skills and desire for competency development into account.* Second, we realized that the variety of environments in which community partners develop competencies carries implications for the process and relevance of competency development of community partners overall. While the enhancement of teaming skills may translate to individual career development opportunities among faculty and clinical research professionals, the diversity among community partners makes it challenging to make any such generalized assumptions. We learned from discussion and interaction with past and current partners that their interest in competency development – individual or teaming – may be associated with the relevance of a potentially community-centric project they are involved with, may be intrinsically inspired, or something else entirely. Additionally, community partners may bring unconventional competencies to the table that are not commonly required in an academic context yet should still be valued. Further, teaming skill development therefore needs to take these individualized needs into account and work with the community partners involved to collaboratively identify the areas for skills development and associated motivation. Whereas community partners may appreciate the opportunity to consider their own strengths in the matrix and may self-identify opportunities to build on their strengths and knowledge, there is not a simple or universal developmental sequence or path for competency development amongst this group.

*Competencies for working with and in community are bi-directional and contextual in nature.* CEnR typically commits to bi-directional learning, where researchers and community partners both expect to share and receive new knowledge and skills throughout the research process [23,24]. The bi-directional nature of working with community partners calls for additional competency development on the side of academic personnel. Here, we chose to further refine the competency of Facilitating Awareness and Exchange and added Welcoming and Inviting, Commitment and Attachment, and Building Genuine Relationships, as additional subcategories. These skills as well as Cognitive Openness and Self-awareness indicate the importance of the skills needed both by community partners and their academic counterparts to build the authentic relationships needed for a fruitful collaboration. The individual competencies facilitating how these relationships are fostered may be transferable to different community settings but the relationships per se are not.

Table 4 below illustrates smart skills in the subcategories Welcoming and Inviting and Commitment and Attachment of the competency domain Facilitating Awareness and Exchange, which are needed by both community partners and academic counterparts.

**Table 4.** Sub-competencies of facilitating awareness and exchange

1.1 Welcoming and inviting *(individual competency)*			
***Defined as:*** *invitational mindset that includes all those with shared interests, based on clarity of objectives and potential co-benefits*			
***Smart skills:***	**Fundamental**	**Skilled**	**Advanced**
**Ability to articulate values of fostering new and sustainable relationships**	*Occasionally articulates value of adding new members or sustaining current members*	*Articulates value of adding new members and sustaining current members*	*Coaches members in articulating value of adding new members and sustaining current members*
**1.2 Commitment and Attachment** (Individual Competency)			
Defined as: making a commitment to long-term relationship and partnership			
***SMART SKILLS:***	***Fundamental***	***Skilled***	***Advanced***
***Desire for the other to succeed rather than compete***	*Make some comments and take positions to show a collaborative spirit*	*Make comments and take positions to show the desire for other to succeed*	*Advocates success of other community partners even at risk of own success*

## Discussion

TS teams are comprised of diverse groups of people at various stages in their career life course, typically from many disciplines. The complex and dynamic nature of TS teams requires describing team science competencies essential to promote ideal team interactions for effective team science. As the three constituency groups worked to describe how team science competencies develop over TS team life course, we realized that the paths towards mastering competencies are unique in each constituency group [20].

For those trainees who are seeking to become faculty, the path of development is quite linear, with some competencies developing before others (Table 2). All competencies are important to learn but there are different levels of expectation for mastery of each competency at different stages of trainees and faculty career lifespan. For example, for undergraduate students, the individual competency of Interdisciplinary Research Management is important for them to learn but does not require a moderate or high level of mastery at that stage, because undergraduate students are still learning. They are not likely to be managing or leading an interdisciplinary research team. As their career progresses and they begin leading research teams, they should gain deeper knowledge and a higher level of mastery of those skills. However, it is important to note that not all competencies were rated at the lowest level of relevance and mastery for undergraduate students. Competencies such as Team-based Communication and Building Trust should be developed to a moderate level rather early in career progression, to help students be effective team members, and deal with other challenges they may face, such as power dynamics among trainees and faculty members. This can lead them to become better mentors and leaders as their career progresses and they become mentors for their future mentees. In summary, the TS teams may include trainees and faculty members at different stages of their careers. The individual and team competencies are relevant for all members, but there are different degrees of relevance and levels of expectation for mastery of each competency, as individuals, as well as teams throughout their lifespan. Mapping the relevance of competencies at each stage of the lifespan identifies specific competencies that could be targeted for learning activities to build team skills at appropriate developmental stages. This information provides additional opportunities to guide the development of team education and training curricula, and competency-based assessment tools for the lifespan of trainees and faculty members [25,26].

For the CRP constituency group, the development of team science competencies does not follow quite the same linear path as for trainees and faculty. Many individuals enter CRP roles as virtual novices to the clinical research workforce; however, they may be those who are transferring from different career roles (e.g., experienced staff nurse becoming a clinical research nurse or recent graduate biology major taking a first clinical research job in data entry). Career moves may move an individual from the experienced level backward to a novice CRP level for clinical research operations, or from recent graduate (novice) as a novice to clinical research. Therefore, interpersonal and team skills learning may depend on education and professional experiences. This phenomenon may also occur when one transitions from one type of clinical research role (e.g., data management) to another (e.g., study coordinating). Because of the diverse nature of CRP roles, progression may be linear; but could bounce backward and forward depending on an individual’s role and career progression. Moreover, team science training has been sidelined to more tangible clinical research operational competencies associated with managing studies, therefore, developing skill sets for communication, leadership, professionalism, and teaming have been less emphasized training objectives. This project serves to create a first-ever published set of leveled team science competencies for CRPs. The three competency levels previously established by the JTF Clinical Trial Competency Framework [14] and further adopted by Duke University perfected their job description and tiered role progression based on the JTF leveling [27,28] and compliments that work which is becoming widely adopted across research sites. Given the global workforce crisis for CRPs [15,29], and even post-doc trainees [30], this work will lead to improvements in training and role expectations that help to strengthen job satisfaction.

The community partner constituency group shifted the paradigm from a linear, developmental model to a more fluid process based on the lived experience, skills, and knowledge already developed by community partners on the team. We expressly rejected any notion of research partners prescribing a training path for any partner and embraced an understanding of the primacy of leadership of the community partner in defining skills/knowledge they brought to and offered the team and any areas of growth they defined for themselves. In doing so, we also asked questions beyond the realm of our brief, including who is inviting, who is joining, who is leading, who is deciding what competencies are relevant, and even who “owns” a competency developed in a team? Such questioning may lead to embracing continued growth in the individual development process among trainees and their mentors. All students and trainees come to their training with different backgrounds and skills. The Individual Development Plan provides an opportunity for individualizing such development within an overall mapping of competencies taking into account the lived experience of the trainee or student [31]. Perhaps community-engaged teams could create a “Team Development Plan” mapped on the framework provided herein, but with the assumption that each member brings different assets and skills and that it is the collective that determines what additional competencies might be important to develop or add with new partners. Existing partners, whether investigators, CRPs, or community members, might provide the mastery and coaching needed to improve overall team competencies.

While the development of team science competencies may be different in the career life course across the three constituencies, we had several light bulb moments that shed light on future work in this area:

1. The three constituencies operate in a complex, interconnected matrix. Due to the complexity of the work done, these interactions are more three-dimensional than two-dimensional and occur simultaneously and energetically as the work overlaps.
2. Awareness that the life course may not be linear for all team members suggests that an adaptive framework could improve teaming in translational team science.
3. Additional research and training in team science is essential for CRPs because research and training in team science has been heretofore lacking.
4. Building trust and humility are important individual and team-related touchpoints that can make or break intentional TS progress.
5. Opportunities for integrating CRPs and community partners in TS teams is a beneficial approach. TS “experience” grows at both the individual and team levels and can have a synergistic impact. It is important for teams to intersect.

Overall, we advocate for a collectivist approach that could shift certain goals from the “individual development plan” model to a “team development plan” model and could be an element of project management planning, leadership, and team charters.
